# Genetic dissection and genomic prediction for pork cuts and carcass morphology traits in pig

**DOI:** 10.1186/s40104-023-00914-4

**Published:** 2023-09-03

**Authors:** Lei Xie, Jiangtao Qin, Lin Rao, Dengshuai Cui, Xi Tang, Liqing Chen, Shijun Xiao, Zhiyan Zhang, Lusheng Huang

**Affiliations:** State Key Laboratory for Pig Genetic Improvement and Production Technology, Jiangxi Agricultural University, Nanchang, 330045 China

**Keywords:** Carcass morphology traits, Genomic selection, Genotype imputation, GWAS, Pork cuts

## Abstract

**Background:**

As pre-cut and pre-packaged chilled meat becomes increasingly popular, integrating the carcass-cutting process into the pig industry chain has become a trend. Identifying quantitative trait loci (QTLs) of pork cuts would facilitate the selection of pigs with a higher overall value. However, previous studies solely focused on evaluating the phenotypic and genetic parameters of pork cuts, neglecting the investigation of QTLs influencing these traits. This study involved 17 pork cuts and 12 morphology traits from 2,012 pigs across four populations genotyped using CC1 PorcineSNP50 BeadChips. Our aim was to identify QTLs and evaluate the accuracy of genomic estimated breed values (GEBVs) for pork cuts.

**Results:**

We identified 14 QTLs and 112 QTLs for 17 pork cuts by GWAS using haplotype and imputation genotypes, respectively. Specifically, we found that *HMGA1, VRTN* and *BMP2* were associated with body length and weight. Subsequent analysis revealed that *HMGA1* primarily affects the size of fore leg bones, *VRTN* primarily affects the number of vertebrates, and *BMP2* primarily affects the length of vertebrae and the size of hind leg bones. The prediction accuracy was defined as the correlation between the adjusted phenotype and GEBVs in the validation population, divided by the square root of the trait's heritability. The prediction accuracy of GEBVs for pork cuts varied from 0.342 to 0.693. Notably, ribs, boneless picnic shoulder, tenderloin, hind leg bones, and scapula bones exhibited prediction accuracies exceeding 0.600. Employing better models, increasing marker density through genotype imputation, and pre-selecting markers significantly improved the prediction accuracy of GEBVs.

**Conclusions:**

We performed the first study to dissect the genetic mechanism of pork cuts and identified a large number of significant QTLs and potential candidate genes. These findings carry significant implications for the breeding of pork cuts through marker-assisted and genomic selection. Additionally, we have constructed the first reference populations for genomic selection of pork cuts in pigs.

**Supplementary Information:**

The online version contains supplementary material available at 10.1186/s40104-023-00914-4.

## Background

Pig carcass cutting is a process of decomposing the post-mortem carcass into various cuts with different sizes and weights according to the tissue structure of different anatomical parts, followed by trimming, cooling, packaging, and preservation. The economic value of pork cuts varies depending on their quantity and quality. Different pork cuts also require diverse cooking and processing methods [1–3]. In recent years, the outbreak of African swine fever in China has issued many policies restricting the transportation of live pigs to prevent the spread of the virus [4, 5], which has presented a new opportunity for the development of chilled meat. Furthermore, due to the rise in living standards and the fast-paced lifestyle, consumers have shifted their pork consumption habits from purchasing hot carcasses for direct cutting and selling to opting for pre-packaged chilled meat that suits their preferences [1, 6]. This further led to the widespread acceptance and adaptation of chilled meat by the majority of consumers. Consequently, many pig companies are rapidly deploying slaughterhouses and expanding their slaughter-processing capabilities within their production chain to optimize the carcass economic value. Mapping and identifying quantitative trait loci (QTLs) for pork cuts will help to breed merit pigs with higher proportion of expensive pre-cut products to increase the overall value of cuts. To the best of our knowledge, there is a lack of reports on QTLs and causal genes that affect pork cuts, as well as investigations into genomic selection or the evaluation of prediction accuracy for pork cuts. The identification of QTLs and investigation of the genetic mechanisms of pork cut attributes serve as the foundation for enhancing the economic value of pork cuts by improving the accuracy of genomic selective breeding.

In this study, 17 pork cuts and 12 carcass morphology traits were measured on 2,012 pigs from four populations genotyped using the CC1 PorcineSNP50K BeadChip (CC1 Chip) [7, 8]. The aim of this study was to identify QTLs affecting proportion of pork cuts to evaluate the accuracy of selection and the feasibility of industrial application. We employed imputation-based whole-genome sequence (WGS) association analysis to uncover potential causal mutations and major genes affecting pork cuts, comparing it with haplotype-based CC1 Chip genotyping data association analysis [9–11]. These results are essential for pig companies who aim to enhance their advantage in the consumer market, core competitiveness, and brand value. Moreover, genetic dissection of pork cuts is vital for understanding carcass composition, which provides critical reference for studying regulatory mechanisms of skeletal and muscle growth and development in different parts of pigs.

## Materials and methods

### Animals, feeding and sampling

A total of 2,012 pigs were randomly sampled from Muyuan Food Co., Ltd. (Henan, China) for pork cut evaluation, as described by Xei et al. [12]. The experimental pigs including 265 Landrace (LR, 95 sows and 170 barrows), 698 Yorkshire (YK, 435 sows and 263 barrows), 689 Landrace × Yorkshire hybrid (LY, 402 sows and 287 barrows), and 258 Duroc × Landrace × Yorkshire hybrid (DLY, 115 sows and 143 barrows). All pigs were raised under consistent feeding environments and nutritional conditions, and they were provided with the same commercial diets and had unrestricted access to water. More details of breeding environment and pedigree family structure were described in our previous study [12]. Each time approximately 100 pigs were randomly selected from 500 to 1,000 market-aged pigs for slaughter testing. A total of 22 batches of pigs were measured for pork cuts and carcass morphology traits (Table S1). These pigs were uniformly slaughtered centrally, following the specifications described in the Operating Procedures of Livestock and Poultry Slaughtering – Pig (GB/T 17236–2019) [13], at an average age of 180 d.

### Phenotypic determination

**Fig. 1:**
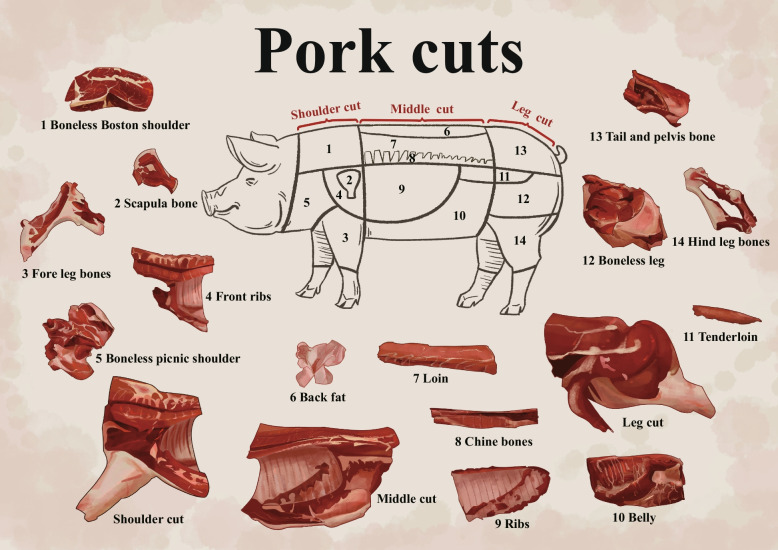
Standardized pork cuts and their corresponding pork cuts in a commercial pig carcass

Twelve carcass morphology traits were measured for all individuals, including carcass straight length (SL), oblique length (OL), thoracic number (THN), lumbar number (LUN), thoracic length (THL), lumbar length (LUL), single lumbar length (SLUL), shoulder backfat depth (SBD), 6th_7th rib backfat depth (RBD), waist backfat depth (WBD), hip backfat depth (HBD), and the mean of backfat depth (MBD). Additionally, the carcass was cut into 17 pork cuts as shown in Fig. 1, and their weight was measured, including three primal cuts (shoulder cut (SC), middle cut (MC), leg cut (LC)), and 14 subprimal cuts (boneless Boston shoulder (BBS), boneless picnic shoulder (BPS), front ribs (FR), fore leg bones (FLB), scapula bone (SB), loin (LO), belly (BE), ribs (RI), chine bones (CB), back fat (BF), boneless leg (BL), tenderloin (TL), hind leg bones (HLB), tail and pelvis bone (TPB)). The determination methods and processes were described in the previously published study [12]. Each pork cut was carefully weighed and measured by the investigators. The proportion of each pork cut was determined through the division of the weight of pork cut by the weight of the entire carcass.

### Genotyping

Genomic DNA was extracted from the muscle tissue of each animal using the routine phenol/chloroform extraction method. Individuals were genotyped using the CC1 PorcineSNP50 BeadChip (51,368 SNPs) [7, 8] according to the manufacturer's protocol. The marker density and accuracy of the CC1 Chip were described in our prior study [7, 8]. Thresholds of individual call rates > 90%, SNPs call rates < 95%, minor allele frequency (MAF) < 5%, and Hardy–Weinberg disequilibrium (*P* < 10^−5^) were filtered out using PLINK (v1.90b6.24) [14]. After quality control, 40,016 SNPs and 2,012 animals were retained for further analysis.

### Imputation of whole-genome sequence variants

Genotype imputation for the experimental population was performed using IMPUTE5 [15] from a high-quality haplotype reference panel including 42,620,918 variants as described by Tong et al. [16]. The haplotype reference panel included whole-genome sequencing data of 1,096 samples from 43 pig breeds (*n* ≥ 3) with an average sequencing depth of 17.1 X. The detailed imputation process was described in our previous study [17]. Variants were called using GATK following the best practice flowchart and were quality controlled by following criteria: (1) SNP: QD < 2.0, QUAL < 30.0, MQ < 40.0, SOR > 3.0, FS > 60.0, MQRankSum <  −2.5, ReadPosRankSum <  −8.0; (2) INDEL: QD < 2.0, QUAL < 30.0, MQ < 40.0, FS > 200.0, ReadPosRankSum <  −20.0. SNPs in the target panel were further filtered with call rate < 95%, or minor allele frequency (MAF) < 5%, or Hardy–Weinberg disequilibrium (*P* < 10E −5) by PLINK (v1.90b6.24) [14]. The haplotypes of the target panel (Sscrofa 11.1) were constructed by SHAPEIT4.2 [18] and PHASEBOOK [19]. Then, genotype imputation was performed between the target and reference panels by IMPUTE5 with default parameters [15]. The imputation accuracy was evaluated by an internal cross-validation solution of IMPUTE5. Specifically, the genotypes of one locus in all individuals in the target panel were masked at a time, and then the masked genotypes were imputed with the haplotype information from the reference panel. The genotypic concordance rate and squared correlation (*R*^2^) between original genotypes from the target panel and imputed genotypes were calculated as imputation accuracies. The accuracies (Mean *R*^2^/concordance rate) of the imputed genotypes for the experimental population were 0.89/99.16%, which implied a high quality of the imputed genotypes.

### Imputation-based of whole-genome sequence GWAS (IGWAS)

Single locus association analysis was conducted using the GEMMA software (version 0.98.1) [20] with a linear mixed model (LMM) that accounts for SNP-based population structure and relatedness between individuals.

$${\varvec{y}}={\varvec{W}}\boldsymbol{\alpha }+{\varvec{X}}{\varvec{\beta}}+{\varvec{u}}+{\varvec{\epsilon}}; {\varvec{u}} \sim {\mathrm{MVN}}_{{n}}\left(0, {{\varvec{G}}\upsigma }_{\mathrm{a}}^{2}\right), {\varvec{\epsilon}} \sim {\mathrm{MVN}}_{{n}}\left(0, {{\varvec{I}}}_{{n}}{\upsigma }_{\mathrm{e}}^{2}\right)$$

where ${\varvec{y}}$ is the vector of phenotypes; ${\varvec{W}}$ is the fixed effect indicator matrix including sex, populations and slaughter batches; $\boldsymbol{\alpha }$ is the corresponding estimations of fixed effects; ${\varvec{X}}$ is incidence matrices of whole-genome imputed SNPs; ${\varvec{\beta}}$ is the SNP substitution effect. ${\varvec{\epsilon}}$ is the residual effect that follows the multivariate normal distribution ${\mathrm{MVN}}_{n}\left(0,{{\varvec{I}}}_{n}{\upsigma }_{\mathrm{e}}^{2}\right)$, in which ${\upsigma }_{\mathrm{e}}^{2}$ is the variance of the residual, ${{\varvec{I}}}_{n}$ is an n-vector of 1s, *n* is the number of phenotypic individuals. The vector ${\varvec{u}}$ is the random polygenic effect that follows the multivariate normal distribution ${\mathrm{MVN}}_{{n}}\left(0, {{\varvec{G}}\upsigma }_{\mathrm{a}}^{2}\right)$, where ${\upsigma }_{\mathrm{a}}^{2}$ is the additive genetic variance and ${\varvec{G}}$ is the kinship matrix calculated using WGS imputed SNPs following VanRaden's method [21] as: ${\varvec{G}}=\frac{{\varvec{M}}{{\varvec{M}^{\varvec{\prime}}}}}{2\sum {p}_{i}(1-{p}_{i})}$, where ***M*** is the allele frequency matrix of centered genotypes with dimensions equal to the number of individuals and the number of SNPs and *p*_*i*_ is the frequency of the reference allele at the *i*-th SNP. Using Bonferroni corrections of 0.05 divided by the number of SNPs to correct multiple comparisons would result in an overly stringent threshold in our study, as many SNPs are highly correlated. Pe'er et al. [22] and Johnson et al. [23] proposed that a genome-wide significance threshold of 5 × 10^−8^ could be used in human GWAS based on independent haplotype blocks in an African population structure. We used the same genome-wide threshold in our study based on the assumption that an equal number of independent haplotype segments exist between pigs and humans. The chromosome-wide significance threshold of 1 × 10^−6^ was used as the suggestive significance threshold [24, 25].

### Haplotype-based CC1 Chip genotyping data GWAS (HGWAS)

Haplotypes of the SNP genotypes were constructed by PHASEBOOK [19]. It assumes that all haplotypes in the population can be traced back to a predetermined number (K = 10) of ancestral haplotypes [26]. Then a hidden Markov model was employed to infer the ancestral haplotypes inherited by each individual at each locus [21]. To detect the association between phenotypes and the haplotype status, a linear mixed framework was used similar to single locus association with a difference in the incidence matrices. In this model, ***X*** is the incidence matrices of the ancestral haplotypes rather than SNP genotypes. The haplotype effects were fitted as random effects. ***G*** is the kinship matrix calculated from the SNP genotypes using VanRaden's method.

### Statistical models to genome prediction

Two genomic selection models were implemented to evaluated the genomic accuracy of pork cuts. (1) Genomic best linear unbiased prediction (GBLUP) [21], which is the most widely used model in genome breeding practice. The mixed linear model is as follows:

$${\varvec{y}}=1{\varvec{\mu}}+{\varvec{W}}\boldsymbol{\alpha }+{\varvec{Z}}{\varvec{a}}+{\varvec{e}}$$

where ${\varvec{y}}$ is the vector of phenotype, ${\varvec{\mu}}$ is the overall mean, $\boldsymbol{\alpha }$ is the fixed effect including sex, populations and slaughter batches, ${\varvec{a}}$ is the vector of genomic breeding values of all individuals, ${\varvec{e}}$ is the vector of residuals, **1** is a vector of ones, ${\varvec{W}}$ is the indicator matrix of $\boldsymbol{\alpha }$, and ${\varvec{Z}}$ is the indicator matrix of ${\varvec{a}}$. Assume that ${\varvec{e}}$ follows a normal distribution of N(0,${\varvec{I}}{\sigma }_{e}^{2}$), and ***a*** follows a normal distribution of N(0*,*${\varvec{G}}{\sigma }_{a}^{2}$). Where ${\upsigma }_{\mathrm{a}}^{2}$ is the additive genetic variance, ***G*** is the kinship matrix obtained from genotype data (included CC1 PorcineSNP50 BeadChip genotype and genome-wide imputed SNPs), which was calculated using VanRaden's method [21], and the detailed calculation method can be found in Yang et al. [27]. Then ${\varvec{a}}$ is solved from the mixed model equations (MME) [28]. In this study, the MME formula is solved by using GCTA software [29], and the estimated genome breeding value of the individual is $\widehat{{\varvec{a}}}$.

(2) Bayesian sparse linear mixed model (BSLMM), which assumes that the effects of markers follow a mixture of two normal distributions [30]. It assumes that all markers have at least a small effect, but some proportion of markers have an additional large effect. The model consists of a standard linear mixed model, with one random effects term, and with sparsity inducing priors on the regression coefficients, corresponding formula is:

$${\varvec{y}}=1{\varvec{\mu}}+{\varvec{W}}\boldsymbol{\alpha }+{\varvec{Z}}\widetilde{{\varvec{\beta}}}+{\varvec{\varepsilon}}$$

$${\varvec{\varepsilon}}\sim {\mathrm{MVN}}_{n}(0,{\tau }^{-1}{\mathbf{I}}_{n})$$

where ${\varvec{y}}$ is the vector of the corrected phenotype, ${\varvec{\mu}}$ is the phenotype mean, $\boldsymbol{\alpha }$ is a vector of the fixed effect including sex, populations and slaughter batches, ${\varvec{W}}$ is the corresponding indicator matrix for $\boldsymbol{\alpha }$**,** ${\varvec{\varepsilon}}$ is the residual effect following a multivariate normal distribution, ${\tau }^{-1}$ is the variance of the residual errors, ${\mathbf{I}}_{n}$ is an n-vector of 1s, *n* is the number of phenotypic individuals. ${\varvec{Z}}$ is the genotype indicator matrix; $\widetilde{{\varvec{\beta}}}$ is the SNP substitution effect vector come from a mixture of two normal distributions:

$$\widetilde{{{\varvec{\beta}}}_{i}}\sim\uppi N\left(0,{{(\sigma }_{a}^{2}+\sigma }_{b}^{2})/p\tau \right)+(1-\pi )N\left(0,{\sigma }_{b}^{2}/p\tau \right)$$

where ${\sigma }_{a}^{2}/p\tau$ is the variance for the SNPs with large effects, ${\sigma }_{b}^{2}/p\tau$ is the variance for the SNPs with minor effects, $p$ is the number of SNPs, and π denotes the proportion of SNPs with large effects. SNP effect $\widetilde{{\varvec{\beta}}}$ was estimated by GEMMA software (version 0.98.1) [20] uses the Markov chain Monte Carlo (MCMC) algorithm and the EBV was calculated as: $GEBV={\sum }_{i=1}^{n}\widetilde{{{\varvec{\beta}}}_{i}} {\times}{SNP}_{i}$, where ${SNP}_{i}$ is the *i*-th SNP genotype of the individual (coded as 0, 1, 2).

### Evaluation of the accuracy of genomic prediction

The accuracy of genomic predictions was evaluated using the fivefold cross-validation method and leave-one-out method. In the fivefold cross-validation method, the population (combined population, YK population or LY population) were divided into five equal groups. For each test, one group of individuals served as the validation dataset, while the other four groups constituted the reference dataset. The test was repeated until all individuals had predicted GEBV, and then the prediction accuracy was calculated. In leave-one-out method, the main step is to take one individual out as the verification group each time, and the remaining individuals as the reference group. The test was repeated to circularly predict the GEBV of all individuals, and calculate the prediction accuracy.

 The prediction accuracy was calculated using the formula proposed by Hayes et al. [31], the formula is as follows:

$$\mathrm{A}={}^{r(GEBV, {y}_{val})}\!\left/ \!{}_{\sqrt{{h}^{2}}}\right.$$

where A is the prediction accuracy, ${y}_{val}$ is the adjusted phenotype of each animal, GEBV is the genomic estimated breeding values, and ${h}^{2}$ is the heritability of the trait. Estimates of heritability (Table S2) for all traits refer to our previous studies [32]. A_p_ and A_w_ denote, respectively, the prediction accuracy of GEBV for the proportion and weight of pork cuts. To further investigate the genomic prediction accuracy impacted by pre-selection of SNPs, we perform GWAS analysis on the reference dataset and selected SNPs which significantly associated with the phenotype to predict the GEBV of individuals in the validation dataset. In the GWAS based on genotype imputation data, we selected the SNPs with *P*-values < 0.01 to predict GEBV. Considering that the SNPs of microarray genotyping are much less than the imputation data, we selected SNPs with *P*-value < 0.05 to predict GEBV in SNP Chip data.

## Results

### Summary of HGWAS

In haplotype-based association studies, we identified a total of 14 QTLs significantly associated with pork cuts and 14 QTLs significantly associated with carcass morphology traits (Table 1). In shoulder cuts, we found three QTLs associated with the proportion of BBS and FLB (Table 1), with the most significant SNP (rs0700815, *P* = 4.03 × 10^−9^) associated with the proportion of FLB located at 31,161,760 bp of *Sus scrofa* chromosome (SSC) 7. This QTL region contains genes (*GRM4*, *HMGA1*, *SMIM29*, *NUDT3* and *PPARD*) associated with body height and limb bone length [33–35]. In middle cuts, we identified 6 QTLs significantly associated with the weight and proportion of RI, BF, and MC (Table 1), with the most significant SNP (rs0702042, *P* = 1.05 × 10^−16^) associated with the RI proportion located at 97,732,109 bp of SSC7. This QTL region contains the *VRTN* gene, which has been identified and functionally validated as a causative gene affecting the number of thoracic vertebrae and ribs [36]. In leg cuts, we found three QTLs significantly associated with the weight and proportion of HLB and LC (Table 1), with the most significant SNP (rs1705050, *P* = 6.25 × 10^−11^) associated with the HLB weight located 188,108 bp downstream of *BMP2* gene on SSC17. In carcass morphology traits, we identified 14 QTLs significantly associated with carcass length (SL, OL), length and number of vertebrae (THL, LUL, THN, LUN, SLUL). The two major candidate genes identified in carcass morphology traits affecting carcass length and vertebral length were *VRTN* and *BMP2*.

**Table 1:** Significant loci associated with pork cuts and carcass morphology traits by haplotypes-based GWAS

Traits	Top SNP	Chr	Pos, bp	*P-*value	Candidate gene^1^	Dis^2^, bp
Pork cuts weight						
Ribs	rs0702042	7	97,732,109	8.41E-13	*VRTN*	107,836
Back fat	rs0103099	1	161,408,832	6.30E-07	*CCBE1*	Within
Hind leg bones	rs1705050	17	15,949,323	6.25E-11	*BMP2*	188,108
Leg cut	rs0501529	5	81,315,221	9.35E-07	*IGF1*	460,749
Pork cuts proportion						
Boneless boston shoulder	rs0701491	7	65,738,048	7.89E-07	*EGLN3*	67,452
Fore leg bones	rs0700815	7	31,161,760	4.03E-09	*HMGA1*	832,351
Fore leg bones	rs0901869	9	84,593,678	8.43E-07	*AGMO*	Within
Ribs	rs0702042	7	97,732,109	1.05E-16	*VRTN*	107,836
Back fat	rs0103099	1	161,408,832	1.57E-07	*CCBE1*	Within
Middle cut	rs0702038	7	97,618,073	1.70E-11	*VRTN*	Within
Middle cut	rs0700525	7	19,581,991	8.72E-07	*GMNN*	4008
Hind leg bones	rs1705090	17	17,801,643	5.32E-08	*BMP2*	2,040,428
Carcass morphology traits						
Straight length	rs1705043	17	15,562,883	2.00E-17	*VRTN*	Within
Straight length	rs0702038	7	97,618,073	1.04E-14	*BMP2*	186,952
Straight length	rs1704946	17	9,342,605	8.71E-07	*IDO2*	Within
Oblique length	rs0702038	7	97,618,073	2.57E-14	*VRTN*	Within
Oblique length	rs1705050	17	15,949,323	4.59E-12	*BMP2*	188,108
Thoracic number	rs0702038	7	97,618,073	6.70E-169	*VRTN*	Within
Thoracic number	rs0701070	7	42,952,142	3.58E-07	*PTCHD4*	182,162
Thoracic length	rs0702038	7	97,618,073	8.16E-60	*VRTN*	Within
Thoracic length	rs1302182	13	103,068,791	2.01E-07	*TMEM266*	Within
Thoracic length	rs1303285	13	159,490,799	3.01E-07	*SI*	1,227,492
Thoracic length	rs0701325	7	56,379,471	4.48E-07	*COL8A1*	116,295
Thoracic length	rs1705043	17	15,562,883	7.04E-07	*BMP2*	186,952
Lumbar length	rs0702035	7	97,347,282	5.84E-07	*VRTN*	267,425
Single lumbar length	rs0101891	1	88,605,020	9.40E-07	*HTR1B*	3,973

^1^Within ± 500 kb of the QTL, the gene closest to the Top SNP or the gene that has been reported to be associated with the phenotype

^2^The distance between the Top SNP site and the candidate gene

Additionally, we detected new QTLs significantly associated with pork cuts, such as a QTL on SSC1 significantly associated with the weight and proportion of BF, with the most significant SNP (rs0700815, *P* = 1.57 × 10^−7^) located at 161,408,832 bp and a QTL on SSC5 associated with LC weight, with the most significant SNP (rs0501529, *P* = 9.35 × 10^−7^) located at the position of 81,315,221 bp, 460,749 bp away from *IGF1* gene.

### Summary of imputation-based IGWAS

Based on imputed genotype data, we identified a total of 167 QTLs significantly related to pork cuts and carcass morphology traits (Table S3). The majority of QTLs identified by HGWAS were also validated in the IGWAS, comprising 54 QTLs associated with weight of pork cuts, 8 QTLs associated with carcass weight, 58 QTLs associated with proportion of pork cuts, and 47 QTLs associated with carcass morphology traits.

In shoulder cuts, a total of 25 QTLs were identified for the weight of pork cuts and 26 QTLs for the proportion of pork cuts (Table 2, Fig. 1, and Table S3). Notably, the largest number of QTLs affecting the weight and proportion of the BBS was observed, with a total of 22 QTLs. The most significant SNP was located at 11,938,089 bp on SSC14, and it was significantly associated with both the weight and proportion of BBS, with *P*-values of 2.75 × 10^−9^ and 1.58 × 10^−9^, respectively. This SNP was located in the intronic region of the *ELP3* gene. In middle cuts, a total of 15 and 19 QTLs were identified affecting the weight and proportion of the pork cuts respectively (Table 2, Fig. 1, Table S3). The QTLs significantly associated with the weight and proportion of CB were the most. The most significant SNP (rs17_15644200) affecting CB weight was located at 15,644,200 bp on SSC17 with a *P*-value of 1.70 × 10^−9^. And rs17_15384749 (15,384,749 bp), located near rs17_15644200, showed a significant association with CB proportion, with a *P*-value of 2.18 × 10^−7^. Both SNPs are situated upstream of the *BMP2* gene. Similarly, the SNPs at positions 97, 130, 183 bp and 97,576,486 bp on SSC7 are top SNPs affecting CB weight and proportion, with *P*-values of 2.88 × 10^−7^ and 3.92 × 10^−9^, respectively. These SNPs are located upstream of the *VRTN* gene at positions 484,524 bp and 38,221 bp. Additionally, two QTLs (97,578,564 – 97,112,240 bp and 97,596,043 – 96,354,619 bp) containing causative gene of *VRTN* affecting vertebra number also significantly affected the weight and proportion of RI. In leg cuts, a total of 14 and 13 QTLs were identified affecting the weight and proportion of the pork cuts respectively. Among them, the greatest number of QTLs that affect the weight and proportion of TL were identified. The most significant SNP affecting the weight of TL was located at 68,490,542 bp on SSC10, with a *P*-value of 8.07 × 10^−8^, located in the intronic region of the *WDR37*. Furthermore, the QTLs significantly associated with HLB weight and proportion were located on SSC17 at 14,621,182–19,590,143 bp and 149,33,905–17,042,539 bp, covering *BMP2*.

**Table 2:** Significant loci associated with pork cuts by imputation-based GWAS

Traits	Top SNP	Chr	Pos, bp	*P*-value	Candidate gene^1^	Dis^2^, bp
Pork cuts weight						
Boneless boston shoulder	4_18049606	4	18,049,606	3.44E-07	*MTBP*	482,396
Boneless boston shoulder	8_28310248	8	28,310,248	5.60E-07	*DTHD1*	236,341
Boneless boston shoulder	10_16027531	10	16,027,531	6.94E-08	*CEP170*	41,008
Boneless boston shoulder	10_2216709	10	2,216,709	8.78E-07		
Boneless boston shoulder	11_65555808	11	65,555,808	1.57E-07	*HS6ST3*	Within
Boneless boston shoulder	13_204134036	13	204,134,036	1.44E-08	*DSCAM*	Within
Boneless boston shoulder	13_16622614	13	16,622,614	1.48E-07	*TGFBR2*	161,877
Boneless boston shoulder	14_11938089	14	11,938,089	2.75E-09	*ELP3*	Within
Boneless boston shoulder	15_44717893	15	44,717,893	4.52E-07	*WWC2*	Within
Fore leg bones	7_106392250	7	106,392,250	8.66E-07		
Fore leg bones	17_15643342	17	15,643,342	3.35E-11	*BMP2*	106,493
Ribs	7_97578564	7	97,578,564	1.69E-10	*VRTN*	36,143
Ribs	17_15688035	17	15,688,035	5.46E-09	*BMP2*	61,800
Chine bones	4_5079579	4	5,079,579	6.61E-07	*FAM135B*	421,559
Chine bones	7_97130183	7	97,130,183	2.88E-07	*VRTN*	484,524
Chine bones	8_32078955	8	32,078,955	9.80E-07	*APBB2*	Within
Chine bones	9_137791545	9	137,791,545	6.29E-08	*GRB10*	1,049,013
Chine bones	12_23208536	12	23,208,536	2.44E-07	*LASP1*	14,172
Chine bones	17_15644200	17	15,644,200	1.70E-09	*BMP2*	105,635
Chine bones	16_6316048	16	6,316,048	5.41E-08	*MYO10*	170,606
Tenderloin	5_57305189	5	57,305,189	1.01E-07	*RERG*	44,188
Tenderloin	9_5248024	9	5,248,024	9.90E-07	*OR51T1*	Within
Tenderloin	10_68490542	10	68,490,542	8.07E-08	*WDR37*	Within
Tenderloin	10_54307891	10	54,307,891	1.52E-07	*PLXDC2*	Within
Tenderloin	11_23214392	11	23,214,392	2.58E-07	*ENOX1*	23,305
Tenderloin	17_61945735	17	61,945,735	3.08E-07	*COL9A3*	129,390
Hind leg bones	17_15643442	17	15,643,442	4.05E-20	*BMP2*	106,393
Pork cuts proportion						
Boneless boston shoulder	1_129573783	1	129,573,783	2.22E-08	*PLA2G4B*	40,385
Boneless boston shoulder	3_128528849	3	128,528,849	6.59E-07	*RNF144A*	218,877
Boneless boston shoulder	4_59046856	4	59,046,856	8.50E-07	*PEX2*	205,481
Boneless boston shoulder	7_79346730	7	79,346,730	5.01E-08	*OR4K17*	1319
Boneless boston shoulder	8_22044470	8	22,044,470	4.74E-07		
Boneless boston shoulder	9_133440994	9	133,440,994	3.37E-08	*LAMB3*	89,797
Boneless boston shoulder	10_16027531	10	16,027,531	4.53E-08	*CEP170*	41,008
Boneless boston shoulder	10_1972864	10	1,972,864	8.34E-07	*RGS18*	433,201
Boneless boston shoulder	13_204136763	13	204,136,763	2.72E-07	*DSCAM*	Within
Boneless boston shoulder	14_11938089	14	11,938,089	1.58E-09	*ELP3*	Within
Boneless boston shoulder	14_2182509	14	2,182,509	8.90E-07	*SYK*	21,337
Boneless boston shoulder	15_45520738	15	45,520,738	1.21E-07	*WWC2*	717,973
Boneless boston shoulder	18_39232070	18	39,232,070	4.89E-07	*BMPER*	180,768
Fore leg bones	5_59607391	5	59,607,391	3.80E-07	*DDX47*	26,034
Fore leg bones	7_30253940	7	30,253,940	9.26E-08	*HMGA1*	66,514
Fore leg bones	17_16478561	17	16,478,561	2.93E-08	*HAO1*	265,879
Ribs	7_97596043	7	97,596,043	1.32E-18	*VRTN*	18,664
Ribs	7_24454624	7	24,454,624	7.83E-07	*NOTCH4*	198,343
Ribs	9_133934063	9	133,934,063	2.87E-07		
Ribs	16_2809427	16	2,809,427	7.97E-07	*DNAH5*	304,852
Chine bones	4_1312897	4	1,312,897	2.14E-07	*LY6L*	12,946
Chine bones	7_97576486	7	97,576,486	3.92E-09	*VRTN*	38,221
Chine bones	7_36885255	7	36,885,255	8.73E-07	*TFEB*	Within
Chine bones	15_10051390	15	10,051,390	8.89E-08	*LRP1B*	Within
Chine bones	17_15384749	17	15,384,749	2.18E-07	*BMP2*	365,086
Tenderloin	4_79051804	4	79,051,804	7.54E-07	*SNAI2*	213,074
Tenderloin	10_54307891	10	54,307,891	2.29E-07	*PLXDC2*	Within
Tenderloin	10_29167522	10	29,167,522	8.24E-07	*GOLM1*	Within
Tenderloin	13_12587359	13	12,587,359	7.12E-07	*TOP2B*	11,776
Hind leg bones	4_10303912	4	10,303,912	4.68E-09	*ASAP1*	Within
Hind leg bones	17_15643251	17	15,643,251	1.60E-10	*BMP2*	106,584

^1^Within ± 500 kb of the QTL, the gene closest to the Top SNP or the gene that has been reported to be associated with the phenotype

^2^The distance between the Top SNP site and the candidate gene

**Fig. 2:**
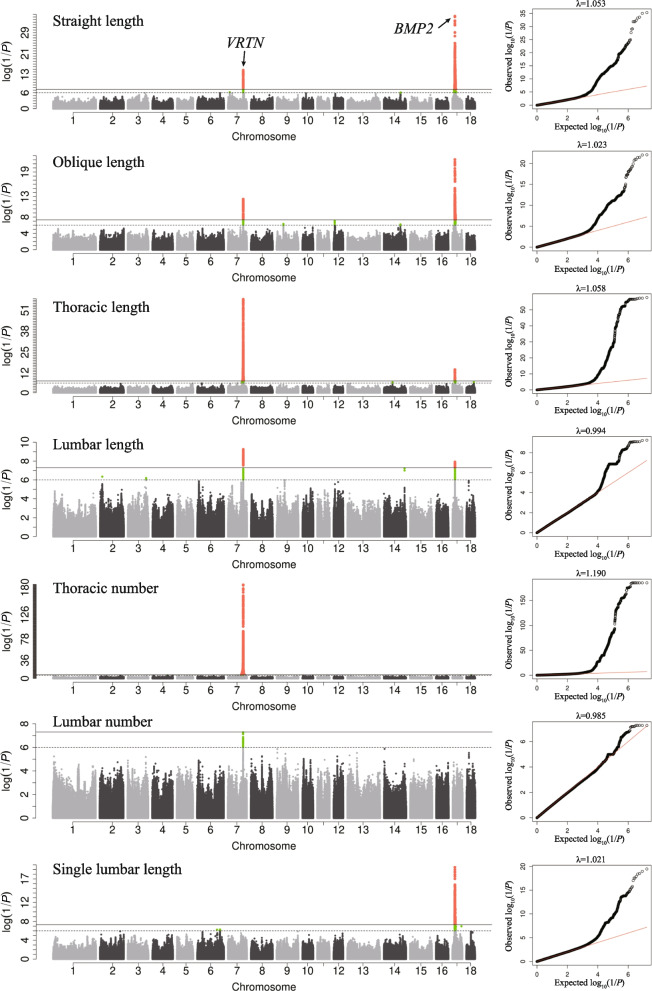
GWAS results of length-related carcass morphology traits. (left) Manhattan plots for carcass morphology traits with the data after imputation. (right) Quantile–quantile plots (Q-Q plots) for carcass morphology traits. In the Manhattan plots, the *y*-axis and *x*-axis represent the −log_10_(*P*-value) of the SNPs and the genomic positions separated by chromosomes, respectively. The tomato puree points represent SNPs that exceeded the genome-wide significance threshold (−log_10_(5 × 10^−8^)). The green points represent SNPs that exceeded the suggestive significance threshold (−log_10_(1 × 10^−6^)). In Q-Q plots, the *y*-axis and *x*-axis represent the expected and observed −log_10_(*P*-value), respectively

In carcass morphology traits, 27 QTLs associated with carcass length and vertebral length, 4 QTLs associated with vertebral number, and 16 QTLs associated with the thickness of backfat were detected (Table 3 and Table S3). Among them, *VRTN* on SSC7 and *BMP2* on SSC17 were found to be the major QTLs affecting carcass length, vertebrae length, and number of vertebrae (Fig. 2). The QTL near to *VRTN* was significantly associated with various traits such as carcass SL, OL, THL, LUL, THN, and LUN and QTL near *BMP2* was also significantly associated with SL, OL, THL, LUL, and SLUL. Interestingly, *VRTN* was found to affect carcass length and total vertebral length by increasing the number of vertebrae, while the *BMP2* may affect these traits by affecting the length of every vertebra. In backfat thickness traits, the most significant SNP was located at 12,758,893 bp on SSC7 with 38,175 bp upstream of the *ATXN1* gene, which was significantly associated with MBD, with a *P*-value of 4.05 × 10^−8^ (Table 3). Furthermore, two QTLs affecting the MBD were identified in the region of 159,644–161,160 kb of SSC1 and 7,347–7,356 kb of SSC2, which affect RBD and WBD (Table 3). The most significant SNPs in these two QTLs were rs1_161160798 (SSC1: 161,160,798 bp) and rs2_7347710 (SSC2: 7,347,710 bp), located at 386,674 bp downstream of *MC4R* and 158,720 bp downstream of *BATF2* gene, and with the *P*-values of 3.45 × 10^−7^ and 1.23 × 10^−7^, respectively.

**Table 3:** Significant loci associated with carcass morphology traits by imputation-based GWAS

Traits	Top SNP	Chr	Pos, bp	*P*-value	Candidate gene^1^	Dis^2^, bp
Straight length	7_97579520	7	97,579,520	2.08E-15	*VRTN*	35,187
Straight length	7_11494001	7	11,494,001	6.08E-07	*JARID2*	Within
Straight length	14_106872034	14	106,872,034	7.89E-07	*SORBS1*	23,729
Straight length	17_11091283	17	11,091,283	5.35E-08	*AP3M2*	75,878
Straight length	17_15692918	17	15,692,918	4.79E-36	*BMP2*	56,917
Straight length	17_21101373	17	21,101,373	4.17E-08	*SPTLC3*	668,111
Oblique length	7_97595573	7	97,595,573	4.07E-13	*VRTN*	19,134
Oblique length	9_43335271	9	43,335,271	4.81E-07	*CADM1*	350,392
Oblique length	12_7316631	12	7,316,631	9.06E-08	*C17orf80*	393,636
Oblique length	14_105368901	14	105,368,901	6.16E-07	*SLC35G1*	23,627
Oblique length	17_15758097	17	15,758,097	8.54E-23	*BMP2*	Within
Thoracic length	7_97595573	7	97,595,573	2.03E-58	*VRTN*	19,134
Thoracic length	14_55238487	14	55,238,487	3.43E-07	*NID1*	1,165
Thoracic length	17_15758097	17	15,758,097	4.71E-15	*BMP2*	Within
Thoracic length	17_19496491	17	19,496,491	1.12E-07	*JAG1*	94,761
Thoracic length	18_47976390	18	47,976,390	2.96E-07	*NPY*	9,335
Lumbar length	2_14043586	2	14,043,586	4.50E-07	*SSRP1*	414,353
Lumbar length	3_117271220	3	117,271,220	6.35E-07	*APOB*	Within
Lumbar length	7_97585410	7	97,585,410	5.53E-10	*VRTN*	29,297
Lumbar length	14_131660585	14	131,660,585	5.99E-08	*TACC2*	Within
Lumbar length	17_15643493	17	15,643,493	1.22E-08	*BMP2*	106,342
Single lumbar length	6_145816008	6	145,816,008	5.12E-07	*SLC35D1*	219,942
Single lumbar length	6_126510205	6	126,510,205	5.53E-07	*PIK3C3*	467,044
Single lumbar length	17_15643442	17	15,643,442	3.36E-20	*BMP2*	106,393
Single lumbar length	17_57495096	17	57,495,096	1.05E-07	*BMP7*	88,995
Single lumbar length	17_21269115	17	21,269,115	1.18E-07	*BTBD3*	390,900
Single lumbar length	17_13713769	17	13,713,769	2.39E-07	*PRNP*	8,451
6th_7th rib backfat depth	1_161160798	1	161,160,798	2.46E-07	*MC4R*	386,674
6th_7th rib backfat depth	1_14679941	1	14,679,941	2.94E-07	*ESR1*	186,578
6th_7th rib backfat depth	2_7347710	2	7,347,710	9.21E-07	*BATF2*	158,720
6th_7th rib backfat depth	7_12752211	7	12,752,211	1.80E-07	*ATXN1*	31,493
Waist backfat depth	1_161834607	1	161,834,607	1.99E-07	*MC4R*	1,060,483
Waist backfat depth	1_238788828	1	238,788,828	9.41E-07	*IGFBPL1*	331,784
Waist backfat depth	2_15159846	2	15,159,846	3.17E-07	*CELF1*	Within
Waist backfat depth	7_77480725	7	77,480,725	3.06E-07	*TRAV3*	1205
Waist backfat depth	14_36628672	14	36,628,672	5.19E-07	*MED13L*	244,110
Hip backfat depth	2_9942614	2	9,942,614	8.14E-07	*SYT7*	5311
Hip backfat depth	4_10303912	4	10,303,912	3.20E-07	*ASAP1*	Within
Hip backfat depth	18_41881878	18	41,881,878	9.71E-07	*GHRHR*	148,632
Mean of backfat depth	1_161160798	1	161,160,798	3.45E-07	*MC4R*	386,674
Mean of backfat depth	2_7347710	2	7,347,710	1.23E-07	*BATF2*	158,720
Mean of backfat depth	7_12758893	7	12,758,893	4.05E-08	*ATXN1*	38,175
Mean of backfat depth	7_9256447	7	9,256,447	9.06E-07	*PHACTR1*	Within

^1^Within ± 500 kb of the QTL, the gene closest to the Top SNP or the gene that has been reported to be associated with the phenotype

^2^The distance between the Top SNP site and the candidate gene

### Accuracy of genomic predictions

**Fig. 3:**
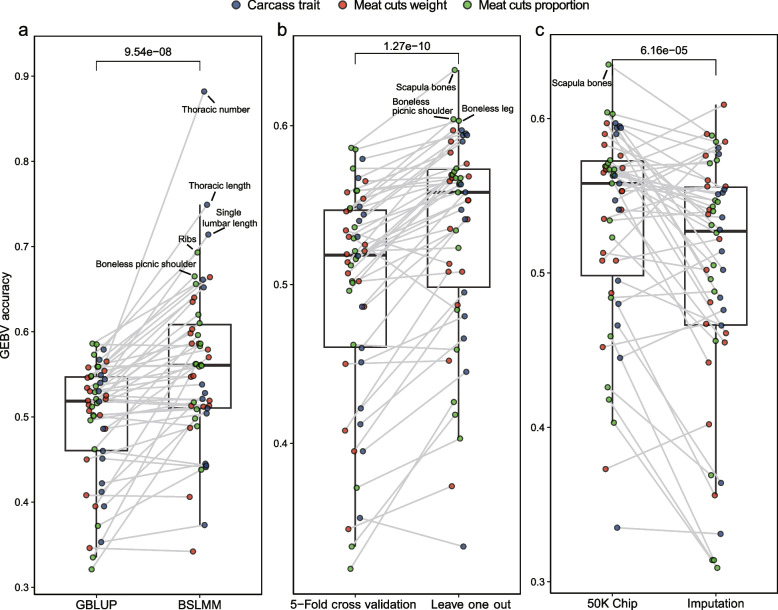
Boxplots comparing the prediction accuracy of GEBV across different models, validation methods, and SNP datasets. **a** Comparison of the accuracy of predicting GEBV using the GBLUP and BSLMM models based on the CC1 Chip genotyping data. **b** Comparison of the accuracy of predicting GEBV using the fivefold cross-validation and leave-one-out method based on the CC1 Chip genotyping data. **c** Comparison of the accuracy of predicting GEBV using the CC1 Chip genotyping data and genotype imputation data based on GBLUP models. CC1 Chip represents CC1 Chip genotyping data. Imputation represents imputation-based of whole-genome sequence

The accuracy of GEBV for all traits using SNP Chip data were presented in Table 4. In pork cuts, the highest prediction accuracy was RI (A_p_ = 0.693, A_w_ = 0.664), followed by BPS (A_p_ = 0.665, A_w_ = 0.640), and the lowest prediction accuracy was TPB (A_p_ = 0.342, A_w_ = 0.438). In carcass morphology traits, the highest prediction accuracy was THN (A = 0.882), followed by LHN (A = 0.749), and the lowest prediction accuracy was LUN (A = 0.373) (Table 4). Additionally, pork cuts and carcass morphology traits with the highest prediction accuracy using the GBLUP model were SB (A_p_ = 0.586, A_w_ = 0.554) and THL (A = 0.579), respectively (Table 4). Importantly, the accuracy of prediction using the BSLMM model was significantly higher than that of the GBLUP model (*P* = 9.54 × 10^−8^) (Fig. 3a), with THN showing the greatest improvement of 0.333. Additionally, we found that the prediction accuracy of the leave-one-out method was significantly higher than that of the fivefold cross-validation method (*P* = 1.27 × 10^−10^) (Fig. 3b).

**Table 4:** Effects of different models, validation methods, and SNPs datasets on the prediction accuracy of GEBV

Genotype data	CC1 chip genotyping data	CC1 chip genotyping data	CC1 chip genotyping data	Genotype imputation data
Models	GBLUP	BSLMM	GBLUP	GBLUP
Validation methods	Fivefold cross-validation	Fivefold cross-validation	Leave-one-out	Leave-one-out
Samples	2,012	2,012	2,012	2,012
Pork cuts weight				
Shoulder cut	0.486	0.570	0.541	0.455
Middle cut	0.519	0.513	0.535	0.556
Leg cut	0.530	0.548	0.567	0.541
Boneless boston shoulder	0.408	0.406	0.508	0.576
Boneless picnic shoulder	0.547	0.640	0.597	0.481
Front ribs	0.395	0.586	0.487	0.356
Fore leg bones	0.502	0.512	0.508	0.522
Scapula bones	0.554	0.579	0.576	0.609
Loin	0.521	0.513	0.553	0.585
Belly	0.525	0.519	0.553	0.556
Ribs	0.565	0.664	0.568	0.461
Chine bones	0.450	0.487	0.452	0.467
Back fat	0.546	0.547	0.565	0.590
Boneless leg	0.558	0.586	0.583	0.560
Tenderloin	0.514	0.603	0.590	0.538
Hind leg bones	0.507	0.635	0.569	0.442
Tail and pelvis bone	0.346	0.342	0.373	0.402
Pork cuts proportion				
Shoulder cut	0.335	0.562	0.418	0.314
Middle cut	0.521	0.610	0.523	0.456
Leg cut	0.559	0.560	0.567	0.546
Boneless boston shoulder	0.496	0.517	0.569	0.571
Boneless picnic shoulder	0.548	0.665	0.604	0.496
Front ribs	0.321	0.498	0.426	0.369
Fore leg bones	0.512	0.562	0.571	0.531
Scapula bones	0.586	0.656	0.635	0.589
Loin	0.502	0.509	0.567	0.543
Belly	0.501	0.489	0.534	0.540
Ribs	0.573	0.693	0.573	0.505
Chine bones	0.462	0.596	0.459	0.314
Back fat	0.536	0.559	0.558	0.547
Boneless leg	0.585	0.583	0.603	0.585
Tenderloin	0.516	0.586	0.563	0.526
Hind leg bones	0.559	0.561	0.564	0.573
Tail and pelvis bone	0.372	0.438	0.403	0.309
Carcass morphology traits				
Half carcass weight	0.530	0.538	0.563	0.551
Carcass weight	0.518	0.521	0.547	0.581
Straight length	0.567	0.661	0.597	0.577
Oblique length	0.540	0.652	0.590	0.528
Thoracic length	0.579	0.749	0.595	0.502
Lumbar length	0.395	0.510	0.445	0.476
Thoracic number	0.549	0.882	0.594	0.484
Lumbar number	0.353	0.373	0.335	0.331
Single lumbar length	0.544	0.714	0.594	0.541
Shoulder backfat depth	0.412	0.528	0.466	0.364
6th_7th rib backfat depth	0.422	0.445	0.495	0.466
Waist backfat depth	0.451	0.441	0.480	0.514
Hip backfat depth	0.460	0.443	0.541	0.536
Mean of backfat depth	0.486	0.504	0.558	0.552

The data in the table are the accuracy of predicting GEBV for all traits

### Different populations, marker densities and pre-selecting markers

**Fig. 4:**
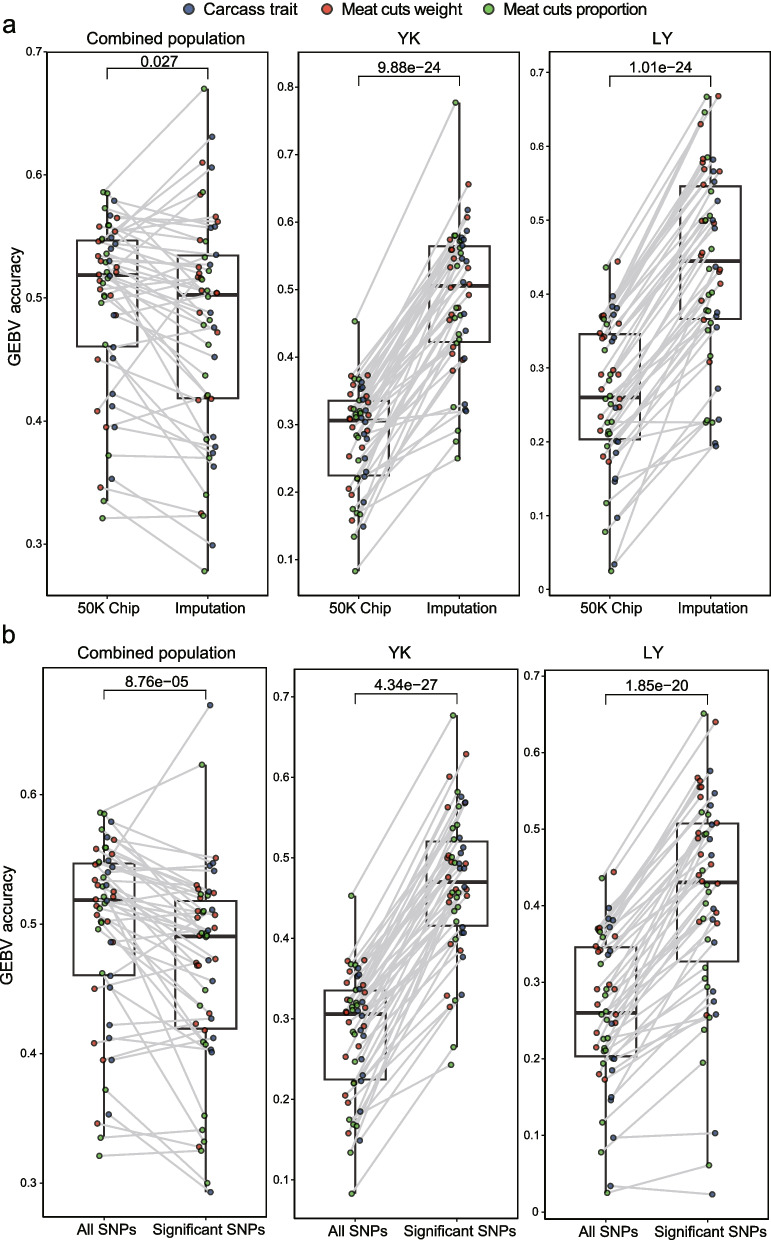
Boxplot comparing the prediction accuracy of GEBV based on different SNP datasets. **a** Comparison of the accuracy of predicting GEBV using the CC1 Chip genotyping data and significant SNPs of IGWAS in different populations. **b** Comparison of the accuracy of predicting GEBV using all SNPs and GWAS significant SNPs of the CC1 chip data in different populations

**Fig. 5:**
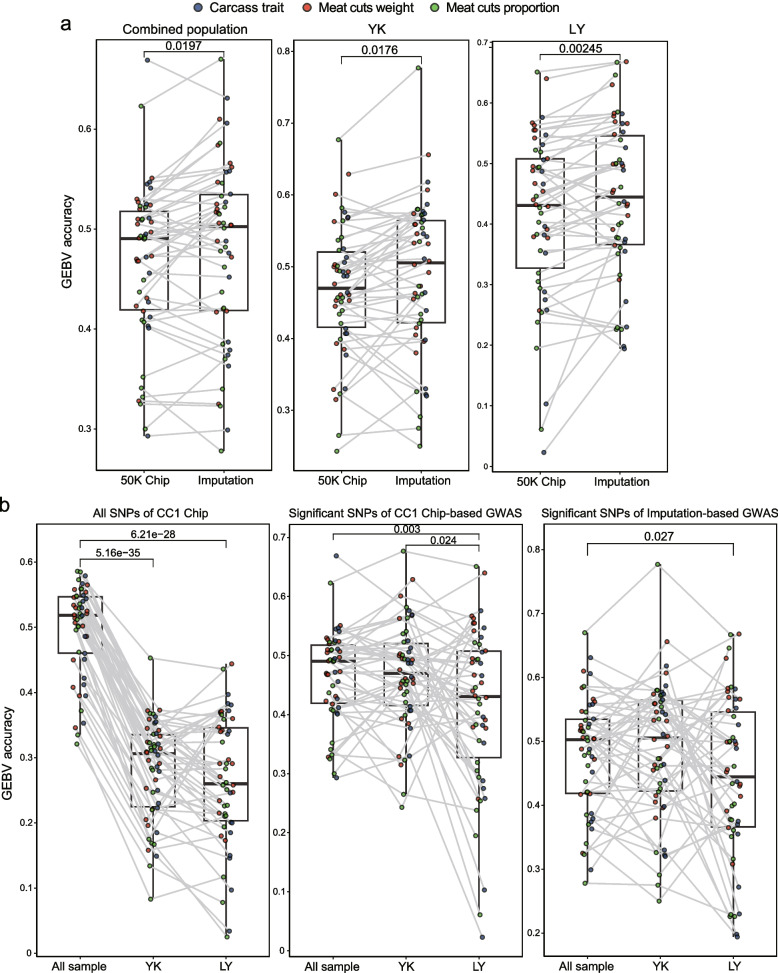
Boxplot comparing the prediction accuracy of GEBV in different populations. Comparison of the accuracy of predicting GEBV using the CC1 chip-based GWAS significant loci data and imputation-based GWAS significant loci in different populations

We found that the prediction accuracy based on the CC1 Chip genotype data was significantly higher than that based on sequence imputation data by GBLUP model (*P* = 6.16 × 10^−5^, Fig. 3c). This shows that the accuracy of the CC1 chip data for genome selection of pork cuts and carcass morphology traits is better. We propose two potential explanations for this result. Firstly, the CC1 Chip, developed collaboratively by the National Laboratory of Pig Genetic Improvement and Breeding Technology and over 12 universities and research institutes in China, includes causal loci that influence body length and weight. Secondly, the poor prediction accuracy of GEBVs based on genotype imputation data may be attributed to the fact that over 98% of imputed genotypes were not associated (*P* > 0.05) with the phenotype, and these loci may be unfavorable to the prediction of GEBV. Previous studies have found that the accuracy of GEBV prediction can be improved by excluding markers that have no effect on traits or have inconsistent effects among different populations [37–39]. Therefore, we pre-selected a set of SNPs to predict GEBV in WGS genotype imputation data. The prediction accuracy of CC1 Chip data was still significantly higher than that of pre-selected genotype imputation data, but the difference in prediction accuracy became smaller (Fig. 4a), with a *P*-value of 0.027, in the combined populations. However, the prediction accuracy of genotype imputation data was significantly higher than that of chip data in the YK and LY populations (Fig. 4a), with significant *P*-values of 9.88 × 10^−24^ and 1.01 × 10^−24^, respectively. Similarly, we chose the GWAS significant loci based on CC1 chip data for genomic prediction. The genomic prediction based on the CC1 Chip data showed that the accuracy of GEBVs for different traits in the combined populations using GWAS significant loci was lower than that using all SNPs (Fig. 4b), with a *P*-value of 8.76 × 10^−5^. However, the prediction accuracy in the YK populations and LY populations was the opposite (Fig. 4b). Furthermore, we compared the prediction accuracy under pre-selection strategy of SNP Chip data and imputation data, we found that the prediction accuracy of imputation-based data was significantly higher than that of the CC1 Chip-based data (Fig. 5a). The results indicate that the selection of GWAS significant loci for GEBV prediction has substantially improved accuracy in single-breed populations, whether using CC1 Chip data or genotype imputation data. However, in the combined population, the prediction accuracy of GEBVs using all markers from CC1 Chip data outperformed others. Also, the prediction accuracy of GEBVs varies significantly across populations when using different datasets (Fig. 5b).

In summary, when predicting GEBVs using genome-wide data, it is advisable to exclude non-relevant loci, also known as pre-selection markers, through GWAS analysis. Different populations may require different strategies for genomic selection.

## Discussion

### Candidate genes affecting body size

We identified three candidate genes associated with skeletal development, namely *VRTN*, *BMP2*, and *HMGA1*. A causal mutation (g.19034 A > C) in *VRTN* was found to be significantly correlated with thoracic vertebra number in our previous studies, and was confirmed by a series of biochemical experiments [36]. In this study, QTLs were also identified in the *VRTN*, which was significantly associated with the weight and proportion of RI and CB, SL, OL, THL and LUL. Li et al. [40] found that the rs320706814 SNP located approximately 123 kb upstream of the *BMP2* was the strongest candidate affecting carcass length. However, this study found that the QTL upstream of the *BMP2* was associated with weight and proportion of FLB, HLB and RI, SL, OL, THL, LUL and SLUL. And, Zhang et al. [35] identified *HMGA1* and *PPARD* as candidate for limb bone length in pigs in the Large White × Minzhu intercross population. Furthermore, other studies have reported that *HMGA1* is a strong candidate gene affecting pig body size [35, 41, 42]. This study found that a QTL in the intron region of the *HMGA1* gene was significantly associated with the proportion of FLB. Overall, *VRTN*, *BMP2*, and *HMGA1* are prominent candidate genes influencing pig body size and play crucial roles in bone development.

### Effects of marker preselection, marker density, and reference population size on genomic prediction

Based on previous research, we know that several factors can influence the accuracy of predicting genomic estimated breeding values (GEBVs). These include the selection and size of the reference population [43, 44], marker density [45, 46], pre-selection of markers [37], prediction models [47–49], and heritability of traits [50, 51]. We compared the effect of different populations on GEBV prediction accuracy and observed significantly higher accuracy in the combined populations when using CC1 Chip data compared to the YK and LY populations (Fig. 5b). This may be due to the limited size of the YK and LY populations, which reduces the accuracy of GEBV prediction. However, using GWAS significant loci for GEBV prediction resulted in significant improvement in accuracy for the YK and LY populations, although it remained lower than that of the combined population. Apart from the reference population size, the variation in linkage disequilibrium between markers in combined populations and single-breed populations also affects prediction accuracy. In the combined population, linkage disequilibrium blocks formed between markers are smaller. Thus, assuming a specific marker has an effect in the combined population, it is more likely due to its higher linkage disequilibrium with the QTL, rather than longer linkage blocks within a single breed. Previous research by Roos et al. [52] also showed that the accuracy of genome prediction is the highest when multiple populations are combined to form a training set, but a higher labeling density is also required. Higher marker density can improve prediction accuracy to some extent, but not all markers will have an impact on traits. In our study, we found that the accuracy of GEBV predictions using genotype imputation data was lower than that based on CC1 Chip genotyping data. However, when using GWAS significant loci to predict GEBVs of different traits, the accuracy of genotype imputation data significantly improved, and in the single-breed population, the accuracy of genotype imputation data was significantly higher than that of CC1 Chip data. It can be seen that while increasing the marker density, we also need to pre-select the markers to improve GEBVs prediction accuracy [37–39].

### Feasibility of genome-based selection for pork cuts

As we all know, the most important thing in animal breeding is to select elite individuals and those are identified as the candidates with high EBVs. One of the widely used molecular breeding methods is marker-assisted selection, which involves identifying QTLs associated with traits of interest and then using models incorporating these QTLs to predict EBV in individuals [50]. In this study, QTLs related to pork cuts were identified, which has important reference value for breeding pork cuts using marker-assisted selection. However, marker-assisted selection has been gradually replaced by molecular breeding methods based on genomic selection in recent years [53–55]. Genomic selection requires establishing a reference population containing phenotype and genotype individuals, evaluating the effect value of each marker on the target phenotype using a suitable model, and then genotyping the individuals that need to be predicted. The GEBVs of each individual are calculated using the estimated marker effect value of the reference population, and individuals are selected and retained based on their GEBVs ranking [56]. This method improves the accuracy of selective breeding and shortens the generation interval. It is especially effective for difficult-to-measure phenotypes and phenotypes with low heritability [57, 58]. In our previous study, we found that most of the pork cuts were medium to high heritability traits. This suggests that breeding for pork cuts using genomic selection may have higher predictive accuracy. In this study, we predicted the GEBVs of pork cuts weight and proportion and found that the prediction accuracy of pork cuts was similar to that of carcass morphology traits, and the accuracy ranged from 0.342 to 0.693. The prediction accuracy of some pork cuts can even reach above 0.65, such as the proportion of RI and BPS. In addition, the pork cuts are the traits of pigs after slaughter, and it is still challenging to predict the weight and proportion of pork cuts through live bodies. Therefore, the use of genomic selection would be a practical way to select elite pigs for pork cuts early in life.

## Conclusion

In this study, we identified 14 QTLs and 112 QTLs associated with 17 pork cuts, as well as candidate genes, using HGWAS and IGWAS for the first time. Our results suggest the independent regulation of skeletal development by several genes across different body parts. Specifically, we identified *HMGA1* as a candidate gene that affects the size of the fore leg bones, *VRTN* as a causal gene that affects the number of vertebral and rib bones and *BMP2* as candidate gene that affects the size of both hind leg bones and fore leg bones, as well as the length of a single vertebral bone. The QTLs and candidate genes we identified have important implications for marker-assisted selection and genome selection. Moreover, we conducted genomic selection of pork cuts and carcass morphology traits in different populations. We found that the prediction accuracy of GEBVs for pork cuts ranged from 0.342 to 0.693, and that the predictive accuracy of several traits, including ribs, boneless picnic shoulder, tenderloin, hind leg bones, and scapula bones, exceeded 0.6. We also found that genomic selection strategy of using BSLMM model, with higher density of effective markers and pre-selecting markers can improve the accuracy of GEBVs. Furthermore, we constructed the first reference populations for genome selection of pork cuts in pigs. These reference populations contain the genetic information of main commercial breeds of Landrace, Yorkshire, and Duroc, which can be directly used for genome selection for most of the commercial pig companies. Overall, our study provides valuable insights into the genetics of pork cuts in pigs and lays a foundation for improving the efficiency of pig breeding programs.

### Supplementary Information

**Additional file 1: Table S1.** Summary information for four populations.

**Additional file 2: Table S2.** Estimates of heritabilities with their standard error (SE) for pork cuts and carcass morphology traits in the combined populations.

**Additional file 3: Table S3.** Significant loci associated with pork cuts and carcass morphology traits by GWAS after genotype imputation.

## Data Availability

The data that support the findings of this study are available from the corresponding author upon reasonable request.

## Abbreviations

BBS: Boneless boston shoulder
BE: Belly
BF: Back fat
BL: Boneless leg
BPS: Boneless picnic shoulder
CB: Chine bones
DLY: Duroc × Landrace × Yorkshire hybrid
EBV: Estimated breed value
FLB: Fore leg bones
FR: Front rib
GEBV: Genomic estimated breed value
GWAS: Genome-wide association study
HBD: Hip backfat depth
HGWAS: Haplotype-based of CC1 Chip genotyping data GWAS
HLB: Hind leg bones
IGWAS: Imputation-based of whole-genome sequence GWAS
LC: Leg cut
LR: Landrace
LO: Loin
LUL: Lumbar length
LUN: Lumbar number
LY: Landrace and Yorkshire hybrid
MBD: Mean of backfat depth
MC: Middle cut
MCP: Meat cut proportion
OL: Oblique length
QTL: Quantitative trait locus
RBD: 6th_7th rib backfat depth
RI: Ribs
SB: Scapula bone
SBD: Shoulder backfat depth
SC: Shoulder cut
SL: Straight length
SLUL: Single lumbar length
SNP: Single nucleotide polymorphism
SSC: Sus scrofa chromosome
THL: Thoracic length
THN: Thoracic number
TL: Tenderloin
TPB: Tail and pelvis bone
WBD: Waist backfat depth
YK: Yorkshire
