# Supplementary material for: Genetic dissection and genomic prediction for pork cuts and carcass morphology traits in pig
Source: J Anim Sci Biotechnol. 2023 Sep 3;14:116. doi: 10.1186/s40104-023-00914-4 (PMC10475202; doi:10.1186/s40104-023-00914-4)
Supplement: Supplementary file 1 — Additional file 1: Table S1. Summary information for four populations. [file 40104_2023_914_MOESM1_ESM.docx]

**Additional file 1: Table S1** Summary information for four populations [1]

| Populations ^1^ | Parental structure | Number ^2^ | Sows ^3^ | Barrows ^4^ | Slaughter batch | Age at slaughter |
| --- | --- | --- | --- | --- | --- | --- |
| LD | 101♀× 50♂ | 278 | 179 | 99 | 3 | 180±3 d |
| YK | 281♀× 120♂ | 742 | 458 | 284 | 8 | 180±3 d |
| LY | 358♀× 165♂ | 725 | 418 | 307 | 8 | 180±3 d |
| DLY | 158♀× 78♂ | 267 | 118 | 149 | 3 | 180±3 d |

^1^ LD: Landrace; YK: Yorkshire; LY: Landrace × Yorkshire or Yorkshire × Landrace; DLY: Duroc × Landrace × Yorkshire

^2^ Number of animals evaluated

^3^ Number of sows evaluated

^4^ Number of barrows evaluated

**References:**

1. Xie L, Qin J, Rao L, Cui D, Tang X, Xiao S, et al. Effects of carcass weight, sex and breed composition on meat cuts and carcass trait in finishing pigs. J Integr Agr. 2023;22:1489–501.
