# Supplementary material for: Genetic dissection and genomic prediction for pork cuts and carcass morphology traits in pig
Source: J Anim Sci Biotechnol. 2023 Sep 3;14:116. doi: 10.1186/s40104-023-00914-4 (PMC10475202; doi:10.1186/s40104-023-00914-4)
Supplement: Supplementary file 2 — Additional file 2: Table S2. Estimates of heritabilities with their standard error (SE) for pork cuts and carcass morphology traits in the combined populations. [file 40104_2023_914_MOESM2_ESM.docx]

**Additional file 2: Table S2** Estimates of heritabilities with their standard error (SE) for pork cuts and carcass morphology traits in the combined populations [1]

| **Trait name** | **Estimates of heritability** ± **SE** |
| --- | --- |
| Number of animals evaluated | 2012 |
| Pork cuts weight |  |
| Shoulder cut | 0.38±0.04 |
| Middle cut | 0.35±0.04 |
| Leg cut | 0.38±0.04 |
| Boneless Boston shoulder | 0.29±0.04 |
| Boneless picnic shoulder | 0.47±0.04 |
| Front ribs | 0.24±0.04 |
| Fore leg bones | 0.34±0.04 |
| Scapula bones | 0.39±0.04 |
| Loin | 0.34±0.04 |
| Belly | 0.35±0.04 |
| Ribs | 0.43±0.04 |
| Chine bones | 0.21±0.04 |
| Back fat | 0.41±0.04 |
| Boneless leg | 0.39±0.04 |
| Tenderloin | 0.35±0.04 |
| Hind leg bones | 0.38±0.04 |
| Tail and pelvis bone | 0.14±0.04 |
| Pork cuts proportion |  |
| Shoulder cut | 0.14±0.03 |
| Middle cut | 0.27±0.04 |
| Leg cut | 0.31±0.04 |
| Boneless Boston shoulder | 0.33±0.04 |
| Boneless picnic shoulder | 0.39±0.04 |
| Front ribs | 0.15±0.04 |
| Fore leg bones | 0.38±0.04 |
| Scapula bones | 0.44±0.04 |
| Loin | 0.36±0.04 |
| Belly | 0.32±0.04 |
| Ribs | 0.37±0.04 |
| Chine bones | 0.23±0.04 |
| Back fat | 0.44±0.04 |
| Boneless leg | 0.34±0.04 |
| Tenderloin | 0.27±0.04 |
| Hind leg bones | 0.38±0.04 |
| Tail and pelvis bone | 0.13±0.04 |
| Carcass morphology traits |  |
| Carcass weight | 0.39±0.05 |
| Half carcass weight | 0.40±0.04 |
| Straight length | 0.45±0.04 |
| Oblique length | 0.45±0.04 |
| Thoracic length | 0.43±0.04 |
| Lumbar length | 0.18±0.04 |
| Thoracic number | 0.48±0.04 |
| Lumbar number | 0.12±0.03 |
| Single lumbar length | 0.24±0.04 |
| Shoulder backfat depth | 0.26±0.04 |
| 6th_7th rib backfat depth | 0.32±0.04 |
| Waist backfat depth | 0.23±0.04 |
| Hip backfat depth | 0.32±0.04 |
| Mean of backfat depth | 0.43±0.04 |

**References**

1. Xie L, Qin J, Yao T, Tang X, Cui D, Chen L, et al. Genetic dissection of 26 meat cut, meat quality and carcass traits in four pig populations. Genet Sel Evol. 2023;55:43. https://doi.org/10.1186/s12711-023-00817-y.
