# Supplementary material for: Genetic dissection and genomic prediction for pork cuts and carcass morphology traits in pig
Source: J Anim Sci Biotechnol. 2023 Sep 3;14:116. doi: 10.1186/s40104-023-00914-4 (PMC10475202; doi:10.1186/s40104-023-00914-4)
Supplement: Supplementary file 3 — Additional file 3: Table S3. Significant loci associated with pork cuts and carcass morphology traits by GWAS after genotype imputation. [file 40104_2023_914_MOESM3_ESM.docx]

**Additional file 3: Table S3** Significant loci associated with carcass cuts and carcass traits by GWAS after genotype imputation

| **Traits** | **Top SNP** | **Chr** | **Pos, bp** | ***P-*value** | **Candidate gene^1^** | **Dis^2^, bp** |
| --- | --- | --- | --- | --- | --- | --- |
| Carcass cuts weight |  |  |  |  |  |  |
| Boneless Boston shoulder | 4_18049606 | 4 | 18,049,606 | 3.44E-07 | *MTBP* | 482,396 |
| Boneless Boston shoulder | 8_28310248 | 8 | 28,310,248 | 5.60E-07 | *DTHD1* | 236,341 |
| Boneless Boston shoulder | 10_16027531 | 10 | 16,027,531 | 6.94E-08 | *CEP170* | 41,008 |
| Boneless Boston shoulder | 10_2216709 | 10 | 2,216,709 | 8.78E-07 |  |  |
| Boneless Boston shoulder | 11_65555808 | 11 | 65,555,808 | 1.57E-07 | *HS6ST3* | Within |
| Boneless Boston shoulder | 13_204134036 | 13 | 204,134,036 | 1.44E-08 | *DSCAM* | Within |
| Boneless Boston shoulder | 13_16622614 | 13 | 16,622,614 | 1.48E-07 | *TGFBR2* | 161,877 |
| Boneless Boston shoulder | 14_11938089 | 14 | 11,938,089 | 2.75E-09 | *ELP3* | Within |
| Boneless Boston shoulder | 15_44717893 | 15 | 44,717,893 | 4.52E-07 | *WWC2* | Within |
| Boneless picnic shoulder | 3_112719684 | 3 | 112,719,684 | 1.78E-08 | *HADHB* | Within |
| Boneless picnic shoulder | 6_145822402 | 6 | 145,822,402 | 2.69E-07 | *SLC35D1* | 226,336 |
| Boneless picnic shoulder | 17_5881261 | 17 | 5,881,261 | 8.18E-08 | *FRG1* | 80,797 |
| Front ribs | 3_9864353 | 3 | 9,864,353 | 1.29E-07 | *DTX2* | 8,582 |
| Arm bones | 7_106392250 | 7 | 106,392,250 | 8.66E-07 |  |  |
| Arm bones | 17_15643342 | 17 | 15,643,342 | 3.35E-11 | *BMP2* | 106,493 |
| Scapula bone | 4_4466298 | 4 | 4,466,298 | 7.69E-07 | *FAM135B* | Within |
| Scapula bone | 4_16431961 | 4 | 16,431,961 | 9.23E-07 | *ZHX2* | Within |
| Scapula bone | 10_15442965 | 10 | 15,442,965 | 3.45E-07 | *PLD5* | Within |
| Scapula bone | 13_197436698 | 13 | 197,436,698 | 1.34E-08 | *ITSN1* | Within |
| Scapula bone | 13_2093329 | 13 | 2,093,329 | 8.93E-07 | *COL6A5* | Within |
| Shoulder cut | 2_130432080 | 2 | 130,432,080 | 7.96E-07 | *PRRC1* | 11,537 |
| Shoulder cut | 2_14045696 | 2 | 14,045,696 | 8.65E-07 | *OR4B1* | 465,248 |
| Shoulder cut | 12_24190870 | 12 | 24,190,870 | 2.58E-07 | *PNPO* | 2,027 |
| Shoulder cut | 15_124578680 | 15 | 124,578,680 | 7.40E-07 | *ACSL3* | Within |
| Shoulder cut | 17_5881261 | 17 | 5,881,261 | 4.57E-07 | *FRG1* | 80,797 |
| Loin | 8_28324376 | 8 | 28,324,376 | 4.78E-07 | *DTHD1* | 250,469 |
| Loin | 17_53065842 | 17 | 53,065,842 | 9.08E-07 | *ATP9A* | Within |
| Belly | 2_30531227 | 2 | 30,531,227 | 5.19E-07 | *KCNA4* | 74,337 |
| Belly | 12_14969745 | 12 | 14,969,745 | 1.63E-07 | *GH1* | 95,164 |
| Ribs | 7_97578564 | 7 | 97,578,564 | 1.69E-10 | *VRTN* | 36,143 |
| Ribs | 17_15688035 | 17 | 15,688,035 | 5.46E-09 | *BMP2* | 61,800 |
| Chine bones | 4_5079579 | 4 | 5,079,579 | 6.61E-07 | *FAM135B* | 421,559 |
| Chine bones | 7_97130183 | 7 | 97,130,183 | 2.88E-07 | *VRTN* | 484,524 |
| Chine bones | 8_32078955 | 8 | 32,078,955 | 9.80E-07 | *APBB2* | Within |
| Chine bones | 9_137791545 | 9 | 137,791,545 | 6.29E-08 | *GRB10* | 1,049,013 |
| Chine bones | 12_23208536 | 12 | 23,208,536 | 2.44E-07 | *LASP1* | 14,172 |
| Chine bones | 17_15644200 | 17 | 15,644,200 | 1.70E-09 | *BMP2* | 105,635 |
| Back fat | 4_4066695 | 4 | 4,066,695 | 6.96E-07 | *FAM135B* | 307,249 |
| Back fat | 14_132047980 | 14 | 132,047,980 | 8.21E-08 | *HTRA1* | Within |
| Middle cut | 9_135947440 | 9 | 135,947,440 | 8.63E-07 | *VWC2* | 34,333 |
| Boneless leg | 4_10303922 | 4 | 10,303,922 | 1.95E-07 | *ASAP1* | Within |
| Boneless leg | 6_156418213 | 6 | 156,418,213 | 9.83E-07 |  |  |
| Boneless leg | 16_6316048 | 16 | 6,316,048 | 5.41E-08 | *MYO10* | 170,606 |
| Tenderloin | 5_57305189 | 5 | 57,305,189 | 1.01E-07 | *RERG* | 44,188 |
| Tenderloin | 9_5248024 | 9 | 5,248,024 | 9.90E-07 | *OR51T1* | Within |
| Tenderloin | 10_68490542 | 10 | 68,490,542 | 8.07E-08 | *WDR37* | Within |
| Tenderloin | 10_54307891 | 10 | 54,307,891 | 1.52E-07 | *PLXDC2* | Within |
| Tenderloin | 11_23214392 | 11 | 23,214,392 | 2.58E-07 | *ENOX1* | 23,305 |
| Tenderloin | 17_61945735 | 17 | 61,945,735 | 3.08E-07 | *COL9A3* | 129,390 |
| Leg bones | 17_15643442 | 17 | 15,643,442 | 4.05E-20 | *BMP2* | 106,393 |
| Tail and pelvis bone | 3_3759865 | 3 | 3,759,865 | 6.95E-07 | *RADIL* | 43,050 |
| Tail and pelvis bone | 4_126002736 | 4 | 126,002,736 | 9.22E-07 | *BARHL2* | 26,074 |
| Tail and pelvis bone | 7_4976539 | 7 | 4,976,539 | 6.05E-07 | *BMP6* | 82,278 |
| Tail and pelvis bone | 7_14068481 | 7 | 14,068,481 | 9.98E-07 | *RNF144B* | Within |
| Carcass weight |  |  |  |  |  |  |
| Half carcass weight | 4_10303912 | 4 | 10,303,912 | 3.81E-07 | *ASAP1* | Within |
| Half carcass weight | 12_24190568 | 12 | 24,190,568 | 7.28E-07 | *PNPO* | 2,329 |
| Half carcass weight | 16_6316048 | 16 | 6,316,048 | 7.61E-07 | *MYO10* | 170,606 |
| Carcass weight | 1_162343867 | 1 | 162,343,867 | 6.73E-07 | *SEC11C* | 574,341 |
| Carcass weight | 3_69211096 | 3 | 69,211,096 | 4.64E-07 | *ACTG2* | 89,747 |
| Carcass weight | 7_11753134 | 7 | 11,753,134 | 6.89E-07 | *DTNBP1* | 4,586 |
| Carcass weight | 10_18345954 | 10 | 18,345,954 | 5.08E-08 | *SMYD3* | Within |
| Carcass weight | 12_14969498 | 12 | 14,969,498 | 2.25E-07 |  |  |
| Carcass cuts proportion |  |  |  |  |  |  |
| Boneless Boston shoulder | 1_129573783 | 1 | 129,573,783 | 2.22E-08 | *PLA2G4B* | 40,385 |
| Boneless Boston shoulder | 3_128528849 | 3 | 128,528,849 | 6.59E-07 | *RNF144A* | 218,877 |
| Boneless Boston shoulder | 4_59046856 | 4 | 59,046,856 | 8.50E-07 | *PEX2* | 205,481 |
| Boneless Boston shoulder | 7_79346730 | 7 | 79,346,730 | 5.01E-08 | *OR4K17* | 1,319 |
| Boneless Boston shoulder | 8_22044470 | 8 | 22,044,470 | 4.74E-07 |  |  |
| Boneless Boston shoulder | 9_133440994 | 9 | 133,440,994 | 3.37E-08 | *LAMB3* | 89,797 |
| Boneless Boston shoulder | 10_16027531 | 10 | 16,027,531 | 4.53E-08 | *CEP170* | 41,008 |
| Boneless Boston shoulder | 10_1972864 | 10 | 1,972,864 | 8.34E-07 | *RGS18* | 433,201 |
| Boneless Boston shoulder | 13_204136763 | 13 | 204,136,763 | 2.72E-07 | *DSCAM* | Within |
| Boneless Boston shoulder | 14_11938089 | 14 | 11,938,089 | 1.58E-09 | *ELP3* | Within |
| Boneless Boston shoulder | 14_2182509 | 14 | 2,182,509 | 8.90E-07 | *SYK* | 21,337 |
| Boneless Boston shoulder | 15_45520738 | 15 | 45,520,738 | 1.21E-07 | *WWC2* | 717,973 |
| Boneless Boston shoulder | 18_39232070 | 18 | 39,232,070 | 4.89E-07 | *BMPER* | 180,768 |
| Boneless picnic shoulder | 7_97617829 | 7 | 97,617,829 | 9.21E-09 | *VRTN* | Within |
| Boneless picnic shoulder | 14_18837504 | 14 | 18,837,504 | 1.44E-07 |  |  |
| Boneless picnic shoulder | 16_19978835 | 16 | 19,978,835 | 5.44E-07 | *SLC45A2* | Within |
| Front ribs | 6_7177226 | 6 | 7,177,226 | 1.89E-07 | *GCSH* | Within |
| Front ribs | 15_129421846 | 15 | 129,421,846 | 4.20E-07 | *SPHKAP* | Within |
| Arm bones | 5_59607391 | 5 | 59,607,391 | 3.80E-07 | *DDX47* | 26,034 |
| Arm bones | 7_30253940 | 7 | 30,253,940 | 9.26E-08 | *HMGA1* | 66,514 |
| Arm bones | 17_16478561 | 17 | 16,478,561 | 2.93E-08 | *HAO1* | 265,879 |
| Scapula bone | 2_125882583 | 2 | 125,882,583 | 4.15E-07 | *SRFBP1* | 306,873 |
| Scapula bone | 4_106553057 | 4 | 106,553,057 | 3.21E-07 | *OLFML3* | 10,393 |
| Scapula bone | 5_24908316 | 5 | 24,908,316 | 9.81E-07 | *SLC16A7* | 33,313 |
| Scapula bone | 14_141742224 | 14 | 141,742,224 | 7.24E-07 | *SYCE1* | 6,360 |
| Scapula bone | 15_50857591 | 15 | 50,857,591 | 6.41E-07 | *UNC5D* | 55,122 |
| Loin | 1_269662233 | 1 | 269,662,233 | 9.74E-07 | *PTPA* | 230,770 |
| Loin | 7_79881531 | 7 | 79,881,531 | 8.49E-07 | *LPCAT4* | 83,936 |
| Loin | 11_65900974 | 11 | 65,900,974 | 3.90E-07 | *HS6ST3* | Within |
| Belly | 2_30531227 | 2 | 30,531,227 | 5.49E-07 | *KCNA4* | 74,337 |
| Belly | 6_11696034 | 6 | 11,696,034 | 9.94E-07 | *TERF2IP* | 318,209 |
| Belly | 7_48389339 | 7 | 48,389,339 | 3.75E-07 | *TBC1D2B* | 460,246 |
| Ribs | 7_97596043 | 7 | 97,596,043 | 1.32E-18 | *VRTN* | 18,664 |
| Ribs | 7_24454624 | 7 | 24,454,624 | 7.83E-07 | *NOTCH4* | 198,343 |
| Ribs | 9_133934063 | 9 | 133,934,063 | 2.87E-07 |  |  |
| Ribs | 16_2809427 | 16 | 2,809,427 | 7.97E-07 | *DNAH5* | 304,852 |
| Chine bones | 4_1312897 | 4 | 1,312,897 | 2.14E-07 | *LY6L* | 12,946 |
| Chine bones | 7_97576486 | 7 | 97,576,486 | 3.92E-09 | *VRTN* | 38,221 |
| Chine bones | 7_36885255 | 7 | 36,885,255 | 8.73E-07 | *TFEB* | Within |
| Chine bones | 15_10051390 | 15 | 10,051,390 | 8.89E-08 | *LRP1B* | Within |
| Chine bones | 17_15384749 | 17 | 15,384,749 | 2.18E-07 | *BMP2* | 365,086 |
| Back fat | 14_132047980 | 14 | 132,047,980 | 6.80E-07 | *HTRA1* | Within |
| Middle cut | 3_98249245 | 3 | 98,249,245 | 2.14E-07 | *PKDCC* | 9,215 |
| Middle cut | 7_97565214 | 7 | 97,565,214 | 1.42E-11 | *VRTN* | 49,493 |
| Middle cut | 18_9486873 | 18 | 9,486,873 | 3.94E-07 | *KDM7A* | 94,511 |
| Boneless leg | 16_32869928 | 16 | 32,869,928 | 3.57E-07 | *FST* | 58,507 |
| Boneless leg | 17_3746717 | 17 | 3,746,717 | 5.13E-07 | *MSR1* | 110,454 |
| Tenderloin | 4_79051804 | 4 | 79,051,804 | 7.54E-07 | *SNAI2* | 213,074 |
| Tenderloin | 10_54307891 | 10 | 54,307,891 | 2.29E-07 | *PLXDC2* | Within |
| Tenderloin | 10_29167522 | 10 | 29,167,522 | 8.24E-07 | *GOLM1* | Within |
| Tenderloin | 13_12587359 | 13 | 12,587,359 | 7.12E-07 | *TOP2B* | 11,776 |
| Leg bones | 4_10303912 | 4 | 10,303,912 | 4.68E-09 | *ASAP1* | Within |
| Leg bones | 17_15643251 | 17 | 15,643,251 | 1.60E-10 | *BMP2* | 106,584 |
| Tail and pelvis bone | 12_31822383 | 12 | 31,822,383 | 1.04E-07 | *TMEM100* | 185,624 |
| Tail and pelvis bone | 17_45884400 | 17 | 45,884,400 | 8.85E-07 | *PTPRT* | 3,578 |
| Leg cut | 9_50493568 | 9 | 50,493,568 | 2.06E-07 | *SCN3B* | 14,630 |
| Leg cut | 10_21144969 | 10 | 21,144,969 | 3.18E-07 | *NEK7* | 20,686 |
| Leg cut | 16_32869928 | 16 | 32,869,928 | 4.40E-08 | *FST* | 58,507 |
| Carcass morphology traits |  |  |  |  |  |  |
| straight length | 7_97579520 | 7 | 97,579,520 | 2.08E-15 | *VRTN* | 35,187 |
| straight length | 7_11494001 | 7 | 11,494,001 | 6.08E-07 | *JARID2* | Within |
| straight length | 14_106872034 | 14 | 106,872,034 | 7.89E-07 | *SORBS1* | 23,729 |
| straight length | 17_11091283 | 17 | 11,091,283 | 5.35E-08 | *AP3M2* | 75,878 |
| straight length | 17_15692918 | 17 | 15,692,918 | 4.79E-36 | *BMP2* | 56,917 |
| straight length | 17_21101373 | 17 | 21,101,373 | 4.17E-08 | *SPTLC3* | 668,111 |
| oblique length | 7_97595573 | 7 | 97,595,573 | 4.07E-13 | *VRTN* | 19,134 |
| oblique length | 9_43335271 | 9 | 43,335,271 | 4.81E-07 | *CADM1* | 350,392 |
| oblique length | 12_7316631 | 12 | 7,316,631 | 9.06E-08 | *C17orf80* | 393,636 |
| oblique length | 14_105368901 | 14 | 105,368,901 | 6.16E-07 | *SLC35G1* | 23,627 |
| oblique length | 17_15758097 | 17 | 15,758,097 | 8.54E-23 | *BMP2* | Within |
| thoracic number | 4_96030181 | 4 | 96,030,181 | 7.59E-07 | *S100A14* | 312 |
| thoracic number | 7_97606621 | 7 | 97,606,621 | 6.76E-186 | *VRTN* | 8,086 |
| thoracic number | 18_31665347 | 18 | 31,665,347 | 3.36E-07 | *FOXP2* | Within |
| lumbar number | 7_97581792 | 7 | 97,581,792 | 5.36E-08 | *VRTN* | 32,915 |
| 6th_7th rib backfat depth | 1_161160798 | 1 | 161,160,798 | 2.46E-07 | *MC4R* | 386,674 |
| 6th_7th rib backfat depth | 1_14679941 | 1 | 14,679,941 | 2.94E-07 | *ESR1* | 186,578 |
| 6th_7th rib backfat depth | 2_7347710 | 2 | 7,347,710 | 9.21E-07 | *BATF2* | 158,720 |
| 6th_7th rib backfat depth | 7_12752211 | 7 | 12,752,211 | 1.80E-07 | *ATXN1* | 31,493 |
| waist backfat depth | 1_161834607 | 1 | 161,834,607 | 1.99E-07 | *MC4R* | 1,060,483 |
| waist backfat depth | 1_238788828 | 1 | 238,788,828 | 9.41E-07 | *IGFBPL1* | 331,784 |
| waist backfat depth | 2_15159846 | 2 | 15,159,846 | 3.17E-07 | *CELF1* | Within |
| waist backfat depth | 7_77480725 | 7 | 77,480,725 | 3.06E-07 | *TRAV3* | 1,205 |
| waist backfat depth | 14_36628672 | 14 | 36,628,672 | 5.19E-07 | *MED13L* | 244,110 |
| hip backfat depth | 2_9942614 | 2 | 9,942,614 | 8.14E-07 | *SYT7* | 5,311 |
| hip backfat depth | 4_10303912 | 4 | 10,303,912 | 3.20E-07 | *ASAP1* | Within |
| hip backfat depth | 18_41881878 | 18 | 41,881,878 | 9.71E-07 | *GHRHR* | 148,632 |
| mean of backfat depth | 1_161160798 | 1 | 161,160,798 | 3.45E-07 | *MC4R* | 386,674 |
| mean of backfat depth | 2_7347710 | 2 | 7,347,710 | 1.23E-07 | *BATF2* | 158,720 |
| mean of backfat depth | 7_12758893 | 7 | 12,758,893 | 4.05E-08 | *ATXN1* | 38,175 |
| mean of backfat depth | 7_9256447 | 7 | 9,256,447 | 9.06E-07 | *PHACTR1* | Within |
| thoracic length | 7_97595573 | 7 | 97,595,573 | 2.03E-58 | *VRTN* | 19,134 |
| thoracic length | 14_55238487 | 14 | 55,238,487 | 3.43E-07 | *NID1* | 1,165 |
| thoracic length | 17_15758097 | 17 | 15,758,097 | 4.71E-15 | *BMP2* | Within |
| thoracic length | 17_19496491 | 17 | 19,496,491 | 1.12E-07 | *JAG1* | 94,761 |
| thoracic length | 18_47976390 | 18 | 47,976,390 | 2.96E-07 | *NPY* | 9,335 |
| lumbar length | 2_14043586 | 2 | 14,043,586 | 4.50E-07 | *SSRP1* | 414,353 |
| lumbar length | 3_117271220 | 3 | 117,271,220 | 6.35E-07 | *APOB* | Within |
| lumbar length | 7_97585410 | 7 | 97,585,410 | 5.53E-10 | *VRTN* | 29,297 |
| lumbar length | 14_131660585 | 14 | 131,660,585 | 5.99E-08 | *TACC2* | Within |
| lumbar length | 17_15643493 | 17 | 15,643,493 | 1.22E-08 | *BMP2* | 106,342 |
| single lumbar length | 6_145816008 | 6 | 145,816,008 | 5.12E-07 | *SLC35D1* | 219,942 |
| single lumbar length | 6_126510205 | 6 | 126,510,205 | 5.53E-07 | *PIK3C3* | 467,044 |
| single lumbar length | 17_15643442 | 17 | 15,643,442 | 3.36E-20 | *BMP2* | 106,393 |
| single lumbar length | 17_57495096 | 17 | 57,495,096 | 1.05E-07 | *BMP7* | 88,995 |
| single lumbar length | 17_21269115 | 17 | 21,269,115 | 1.18E-07 | *BTBD3* | 390,900 |
| single lumbar length | 17_13713769 | 17 | 13,713,769 | 2.39E-07 | *PRNP* | 8,451 |

^1^Within ±500 kb of the QTL, the gene closest to the Top SNP or the gene that has been reported to be associated with the phenotype

^2^The distance between the Top SNP site and the candidate gene
